# Application of the Milan and IAC–IARC–WHO Systems in diagnosing soft tissue and bone tumors of the salivary glands: The Institut Curie experience

**DOI:** 10.1002/cncy.70040

**Published:** 2025-08-20

**Authors:** Adam Kowalewski, Jędrzej Borowczak, Hervé J. Brisse, Olivier Choussy, Jerzy Klijanienko

**Affiliations:** ^1^ Department of Pathology and Theranostics Institut Curie PSL University Paris France; ^2^ Department of Tumor Pathology Oncology Centre Prof. Franciszek Łukaszczyk Memorial Hospital Bydgoszcz Poland; ^3^ Faculty of Medicine Bydgoszcz University of Science and Technology Bydgoszcz Poland; ^4^ Clinical Department of Oncology Oncology Centre Prof. Franciszek Łukaszczyk Memorial Hospital Bydgoszcz Poland; ^5^ Department of Radiology Institut Curie PSL University Paris France; ^6^ Department of Head and Neck Surgery Institut Curie PSL University Paris France

**Keywords:** fine‐needle aspiration, IAC–IARC–WHO, mesenchymal tumors, Milan System, MSRSGC, risk of malignancy, salivary gland, soft tissue

## Abstract

**Background:**

Soft tissue and bone tumors of the salivary gland were compared using classic 4‐tier, Milan System for Reporting Salivary Gland Cytopathology (MSRSGC), and the International Academy of Cytology‐International Agency for Research on Cancer‐World Health Organization (IAC–IARC–WHO) Reporting System for Soft Tissue Cytopathology.

**Methods:**

The authors retrospectively analyzed 2219 salivary gland fine‐needle aspiration (FNA) samples collected at the Institut Curie in Paris between 1954 and 2022. A total of 86 cases (3.9%) were identified as soft tissue and bone tumors, with histological follow‐up in 63 cases (73%). Cytology was classified according to the classic European 4‐tier system, the MSRSGC, and the IAC–IARC–WHO System.

**Results:**

According to the MSRSGC, eight cases were classified as nonneoplastic, 30 as benign, one as salivary gland neoplasm of uncertain malignant potential, five as suspicious for malignancy, and 42 as malignant. Benign or malignant nature was accurately assessed in 78 cases (90.7%), with exact histologic‐cytologic concordance in 51 cases (59.3%). FNA correctly identified 42 of 44 malignant tumors (95.5%), with exact diagnostic matches in 30 cases (68.2%). The MSRSGC achieved 88.3% overall accuracy, 93.6% sensitivity, and 82.7% specificity. Risk of malignancy (ROM) was comparable for malignant tumors across the classic European system, the MSRSGC, and the IAC–IARC–WHO System, with minor discrepancies observed in benign and indeterminate categories. No cases were assigned as nondiagnostic, atypia of undetermined significance, atypical.

**Conclusions:**

Despite their rarity, soft tissue and bone tumors of the salivary gland can be effectively diagnosed using the MSRSGC, with a high accuracy achieved for malignant cases. Minor discrepancies in ROMs were observed between specific categories of the classic European system, the MSRSGC, and the IAC–IARC–WHO System.

## Abstract 2

Soft tissue and bone tumors of the salivary gland were compared using classic 4‐tier, Milan System for Reporting Salivary Gland Cytopathology (MSRSGC), and the International Academy of Cytology‐International Agency for Research on Cancer‐World Health Organization (IAC–IARC–WHO) Reporting System for Soft Tissue Cytopathology. Despite their rarity, soft tissue and bone tumors of the salivary gland can be effectively diagnosed using the MSRSGC, with a high accuracy achieved for malignant cases. Minor discrepancies in ROMs were observed between specific categories of the classic European system, the MSRSGC, and the IAC–IARC–WHO System.

## INTRODUCTION

Salivary gland neoplasms are typically diagnosed using fine‐needle aspiration (FNA), allowing for pre‐surgical evaluation. In the past decade, various efforts have been made to harmonize cytology reporting in salivary gland lesions. Up to 2018, a 4‐tier system (not significant, benign, suspicious, and malignant) was practiced in continental Europe, whereas a 5‐tier system (not significant, benign, atypical, suspicious, and malignant) was adopted in other regions. Since 2018, the Milan System for Reporting Salivary Gland Cytopathology (MSRSGC) has become the reporting standard^1^, ^2^ and its utility has been confirmed worldwide in both adults and in children.^3^ The standardized description of cytological findings has enabled risk‐based categorization of samples and enabled management strategies based on the assigned categories.^2^

Despite its clinical utility, the MSRSGC has been validated primarily for diagnosing common, predominantly epithelial salivary gland neoplasms. Its efficacy in classifying tumors of other origins remains largely unexplored.^3^ More recently, the International Academy of Cytology (IAC) and the International Agency of Research on Cancer (IARC) initiated the series of IAC–IARC–WHO Cytopathology Reporting Systems, intended to align with the WHO Classification of Tumors (“blue‐books”) proposed to harmonize reporting in soft tissue cytopathology.^4^ To date, only one report based on a large series of soft tissue tumors has been published.^5^

Soft tissue and bone tumors of the salivary gland are exceedingly rare, comprising 1.9% to 5% of all salivary gland tumors.^6^, ^7^, ^8^ Because of their low prevalence and histological diversity, they present a significant diagnostic challenge. These tumors are usually diagnosed based on respective WHO classifications, with categories distinct from those used for salivary gland neoplasms.^9^, ^10^ Consequently, the diagnostic accuracy of the MSRSGC for these tumors remains uncertain, and the applicability of its cytological criteria is unclear.^2^

As cytology reporting for salivary tumors and soft tissue tumors enters a new era, we analyzed the clinical utility the classic 4‐tier European system, the MSRSGC, and the IAC–IARC–WHO Systems for diagnosing primary and secondary soft tissue and bone neoplasms involving the salivary glands.

## MATERIALS AND METHODS

### Sample acquisition and review

A review was conducted on 2219 salivary gland samples collected via FNA at the Institut Curie in Paris from 1954 to 2022. We identified 86 soft tissue and bone tumor samples, which were selected for further analysis. All FNAs were performed by surgical pathologists. Because of the extensive period covered by this study, detailed information on the sampling techniques for each case was not consistently available. In older cases, FNAs were typically performed under palpation guidance. From 1995 onward, most pediatric cases were sampled under ultrasound guidance with radiological assistance, and these procedures were usually performed under general anesthesia.

On inclusion, all cytology specimens were reevaluated and classified according to the classic European 4‐tier system (not significant, benign [BN], suspicious, and malignant [M]),^11^ the second edition of the MSRSGC guidelines (nondiagnostic [ND], nonneoplastic [NN], atypia of undetermined significance [AUS], benign neoplasm [BN], Salivary gland neoplasm of uncertain malignant potential [SUMP], suspicious for malignancy [SM], malignant [M]),^12^ and the IAC–IARC–WHO Reporting System for Soft Tissue Cytopathology (insufficient/inadequate/non‐diagnostic [ND], benign [BN], atypical, neoplasm of uncertain malignant potential [NUMP], suspicious for malignancy [SM], and malignant [M]).^5^

Additionally, although salivary gland myoepitheliomas are classified by the WHO as epithelial neoplasms, they were included in this analysis due to their mesenchymal‐like cytological appearance, which frequently overlaps with that of soft tissue tumors on FNA specimens.

In this study, the “not significant” category—used in the classic 4‐tier European system—was applied to samples with low cellularity or lacking diagnostically relevant findings. Although this system was never formally standardized, our application of this category aligns with what is now defined as “Non‐Diagnostic” or, in some cases, “Non‐Neoplastic” in the MSRSGC. Cytologic adequacy was judged according to MSRSGC criteria; adequate samples with normal salivary gland elements and no atypia were classified as nonneoplastic (category II). Immunocytochemistry (ICC) was performed in selected cases based on clinical indication; however, immunostain selection was not standardized, and detailed ICC data were not systematically recorded due to the retrospective nature of the cohort.

All corresponding histological sections were reviewed and reclassified using the WHO 5th edition terminology.^11^, ^13^ This was especially necessary for older cases diagnosed before 1995.

### Sample staining

Cytological specimens were predominantly stained using the classic May–Grünwald–Giemsa method, with occasional application of the Papanicolaou stain. In recent years, Diff‐Quik staining (ROSE technique) has also been employed to ensure FNA quality. Additionally, many recent cases used cell blocks for immunocytochemistry. Although routine cytological assessment relied primarily on conventional staining techniques, cell blocks were used for immunocytochemistry when diagnostically indicated. However, the assignment of Milan categories relied strictly on cytomorphological features, following the second edition of the MSRSGC criteria.^12^

Histological examinations were performed on slides obtained from corresponding core needle biopsies, surgical biopsies (especially in older cases), and surgical specimens, which were stained with hematoxylin, eosin, and saffron (HES). Starting in the late 1990s, molecular techniques became more available and were employed in selected cases if needed to refine the final diagnosis. Where available, these data contributed to final cytological or histopathological diagnoses.

### Inclusion and exclusion criteria

All samples with soft tissue or bone tumors, either constituting a cytological or final diagnosis, were included in this analysis. For descriptive purposes and categorization, cases were included regardless of histological follow‐up. Further statistical analysis included only cases with available histopathological follow‐up. Benign or nonneoplastic findings diagnosed by FNA without corresponding histology were included if there was reliable clinical follow‐up and radiological assessment. Ultimately, 86 cases (including primary tumors, recurrences, and metastases) were eligible for this analysis. Tumors classified as “intermediate” (ICD‐O code 1) were assigned to the benign or malignant group based on their metastatic potential, following WHO guidelines.^13^ Cytological diagnoses were extracted from the original clinical reports, and all available clinicopathologic data (age, sex, tumor localization, and final histopathological diagnosis) were retrieved from hospital records.

### Statistical analysis

Statistical analysis was performed using STATISTICA 13.3 and Microsoft Excel 2021. For calculating predictive values of FNAs, categories SM and M were classified as “positive” cytopathological tests. Conversely, NN and BN were categorized as “negative” tests for diagnosing malignancy. Our cohort did not include any ND, AUS, or atypical samples. The only SUMP/STNUMP sample was also excluded from the calculation due to its indeterminate malignant potential. Predictive values were reported with their respective 95% confidence intervals (CIs).

## RESULTS

### General findings

Of 86 identified salivary gland soft tissue and bone tumors, 42 (48.8%) were benign and 44 (51.2%) were malignant. The risk of soft tissue or bone malignancy among salivary gland tumors was 1.98% (44 of 2219 cases). The majority of these tumors (66 of 86, 76.7%) originated from the parotid gland, followed by the submandibular gland (14 of 86, 16.3%) and unspecified sites of origin (six of 86, 7%) (Table 1). The median age of patients was 34 years (range, 0.2–88 years), with patients with malignant salivary tumors being significantly younger than those with benign salivary neoplasms (median age, 21 vs. 42 years).

**TABLE 1:** The distribution of soft tissue and bone tumors of the salivary glands.

Site	No. of cases (%), *n* = 86	Benign, No. (%), *n* = 42	Malignant, No. (%), *n* = 44
Parotid gland	66 (76.7)	32 (48.5)	34 (51.5)
Submandibular gland	14 (16.3)	8 (57.1)	6 (42.9)
Not specified sites	6 (7)	2 (33.3)	4 (66.7)

All diagnosed tumors were classified into subgroups according to the WHO Classification of Soft Tissue Tumors (Table 2).^13^ Skeletal muscle tumors were the largest group, comprising 18 of 86 cases (20.9%), with rhabdomyosarcoma, both primary and metastatic, making up the majority (13 primary and four metastatic). Lipoma was the only diagnosed adipocytic tumor and the most common soft tissue tumor in the entire cohort, representing 16 of 86 cases (16.8%). Schwannoma was the most prevalent peripheral nerve sheath tumor, accounting for four of eight cases (50%), whereas fibromatosis constituted 37.5% (three of eight) of fibroblastic and myofibroblastic tumors. Among vascular tumors, there were three hemangiomas (75%) and one angiosarcoma (25%). Metastatic leiomyosarcoma was the only smooth muscle tumor identified (three of three, 100%). Metastatic extraskeletal osteosarcoma and myopericytoma, with two cases each, were the only tumors in the chondro‐osseous and pericytic groups, respectively.

**TABLE 2:** Diagnosed cases categorized by the 2020 WHO Classification of Soft Tissue Tumors.

Category	Malignant potential	No. of cases (%)
Adipocytic		16 (18.6)
Lipoma	Benign	16
Fibroblastic and myofibroblastic		8 (9.3)
Fibromatosis^a^	Locally aggressive	3
Nodular fasciitis	Benign	2
Fibrosarcoma	Malignant	1
Dermatofibrosarcoma protuberans^a^	Uncertain potential, rarely metastasizing	1
Fibroma	Benign	1
Vascular tumors		4 (4.7)
Hemangioma	Benign	3
Angiosarcoma	Malignant	1
Pericytic tumors		2 (2.3)
Myopericytoma	Benign	2
Smooth muscle tumors		3 (3.5)
Metastatic leiomyosarcoma	Malignant	3
Skeletal muscle tumors		18 (20.9)
Metastatic rhabdomyosarcoma	Malignant	13
Primary rhabdomyosarcoma	Malignant	4
Granular rhabdomyoma^a^	Uncertain potential	1
Chondro‐osseous tumors		2 (2.3)
Metastatic extraskeletal osteosarcoma	Malignant	2
Peripheral nerve sheath tumors		7 (8.1)
Schwannoma	Benign	4
Neurofibroma	Benign	1
Neuroma	Benign	1
Malignant peripheral nerve sheath tumor	Malignant	1
Tumors of uncertain differentiation		13 (15.1)
Myoepithelioma^a^	Uncertain potential, rarely metastasizing	5
Sarcoma, NOS	Malignant	5
Metastatic sarcoma, NOS	Malignant	3
Not categorized tumors		15 (17.4)
Metastatic neuroblastoma	Malignant	4
Metastatic chordoma	Malignant	4
Adamantinoma^a^	Uncertain potential, locally aggressive	3
Paraganglioma	Benign	2
Metastatic chondrosarcoma	Malignant	1
Vascular hamartoma	Benign	1

Abbreviations: NOS, not otherwise specified; WHO, World Health Organization.

^a^ Tumors classified as “intermediate” (ICD‐O code 1) by WHO were assigned to the benign or malignant group based on clinical behavior and metastatic potential. Nonmetastasizing tumors (fibromatosis, myoepithelioma, and granular rhabdomyoma) were included among benign neoplasms, whereas metastasizing tumor (dermatofibrosarcoma protuberans and adamantinoma) were grouped with malignant tumors.

Notably, tumors of uncertain differentiation and those not categorized constituted a significant portion of our cohort, representing 32.5% of all diagnosed soft tissue and bone salivary gland tumors. Among these, myoepithelioma and primary sarcoma of the salivary gland were the most common, each accounting for 38.5% (five of 13 cases). This was followed by metastatic sarcoma, which constituted 23% (three of 13 cases).

Using the classic 4‐tier European system we classified 38 cases BN, six as suspicious/inconclusive, and 48 as M (Table 3). None of our cases met the criteria for “not significant.” Cytopathological examination provided an ultimate diagnosis in 23 cases (26.7%), including 10 benign neoplasms (26.3%) and 13 malignancies (31.0%). Of 86 cases, 63 (73.3%) had histological follow‐up, with 32 confirmed as benign and 31 as malignant. Among the benign category, two of 28 samples (7.1%) were malignant, whereas one of six (16.7%) was malignant in the suspicious/inconclusive category. Notably, 28 of 29 samples (96.6%) in the M category were malignant.

**TABLE 3:** Distribution of fine‐needle aspiration biopsies by the classic European system categories and histopathological follow‐up (*n* = 86).

Classic system	Not significant	Benign, No. (%)	Suspicious/inconclusive, No. (%)	Malignant, No. (%)	Total, No. (%)
No. of cases	—	38 (44.2)	6 (7)	42 (48.8)	86 (100)
No. of cases with histological follow‐up	—	28 (73.7)	6 (100)	29 (69)	63 (73.3)
Nonneoplastic	—	0 (0)	0 (0)	0 (0)	0 (0)
Benign neoplasm	—	26 (92.9)	5 (83.3)	1 (3.4)	32 (53.2)
Malignant	—	2 (7.1)	1 (16.7)	28 (96.6)	31 (46.8)

The initial assessment using the MSRSGC classified eight cases as NN, 30 as BN, one as SUMP, five as SM, and 42 as M (Table 4). None of our cases met the criteria for ND or AUS. All BN (30 of 30) and SUMP (one of one) cases were benign. Histological follow‐up was available for 20 of the 30 BN cases, and all confirmed a benign diagnosis; the remaining cases were not histologically evaluated. Among the NN category, two of eight samples (25.0%) were malignant, whereas one of five (20%) was malignant in the SM category. A total of 28 of 29 samples (96.6%) were malignant in the M category.

**TABLE 4:** Distribution of fine‐needle aspiration biopsies by Milan System diagnostic categories and histopathological follow‐up (*n* = 86).

Milan category	ND	NN, No. (%)	AUS	BN, No. (%)	SUMP, No. (%)	SM, No. (%)	M, No. (%)	Total, No. (%)
No. of cases	—	8 (9.3)	—	30 (34.9)	1 (1.2)	5 (5.8)	42 (48.8)	86 (100)
No. of cases with histological follow‐up	—	8 (100)	—	20 (66.7)	1 (100)	5 (100)	29 (69)	63 (73.3)
NN	—	0 (0)	—	0 (0)	0 (0)	0 (0)	0 (0)	0 (0)
BN	—	6 (75)	—	20 (100)	1 (100)	4 (80)	1 (3.4)	32 (53.2)
M	—	2 (25)	—	0 (0)	0 (0)	1 (20)	28 (96.6)	31 (46.8)

Abbreviations: AUS, atypia of undetermined significance; BN, benign neoplasm; M, malignant; ND, nondiagnostic; NN, nonneoplastic; SM, suspicious for malignancy; SUMP, salivary gland neoplasm of uncertain malignant potential.

The following analysis using the IAC–IARC–WHO Cytopathology Reporting Systems brought similar results to the classic 4‐tier European system—8 cases were classified as BN, six as SM, and 48 as M (Table 5). None of our cases met the criteria for the ND and AUS categories. In the BN category, two of 28 samples (7.1%) were malignant, whereas one of six (16.7%) was malignant in the SM category. A total of 28 of 29 samples (96.6%) in the M category were malignant.

**TABLE 5:** Distribution of fine‐needle aspiration biopsies by the IAC–IARC–WHO System categories and histopathological follow‐up (*n* = 86).

IAC–IARC–WHO categories	ND	BN, No. (%)	AUS	STNUMP, No. (%)	SM, No. (%)	M, No. (%)	Total, No. (%)
No. of cases	—	38 (44.2)	—	1 (1.2)	5 (5.8)	42 (48.8)	86 (100)
No. of cases with histological follow‐up	—	28 (73.7)	—	1 (100)	5 (100)	29 (69)	63 (73.3)
NN	—	0 (0)	—	0 (0)	0 (0)	0 (0)	0 (0)
BN	—	26 (92.9)	—	1 (100)	4 (80)	1 (3.4)	32 (53.2)
M	—	2 (7.1)	—	0 (0)	1 (20)	28 (96.6)	31 (46.8)

Abbreviations: AUS, atypia of undetermined significance; BN, benign neoplasm; IAC–IARC–WHO, International Academy of Cytology‐International Agency for Research on Cancer‐World Health Organization; M, malignant; ND, nondiagnostic; NN, nonneoplastic; SM, suspicious for malignancy; STNUMP, soft tissue neoplasm of uncertain malignant potential; SUMP, salivary gland neoplasm of uncertain malignant potential.

The calculated risk of malignancy (ROM) varied slightly between the classic European system, the Milan System, and the IAC–IARC–WHO System. It reached 7.1% for the BN categories in both the classic and IAC–IARC–WHO Systems, but was 25.0% in the NN category and 0% in the BN category of the Milan System. In the suspicious or inconclusive categories, the ROM was 16.7% in the classic system and 20.0% in both the Milan System and the IAC–IARC–WHO System (SM categories), whereas 0% ROM was observed in the SUMP and ST‐NUMP categories. The ROM in the M category was consistent across all three systems, reaching 96.6%. No cases were assigned to the nondiagnostic, AUS, or atypical categories in this cohort.

**FIGURE 1:**
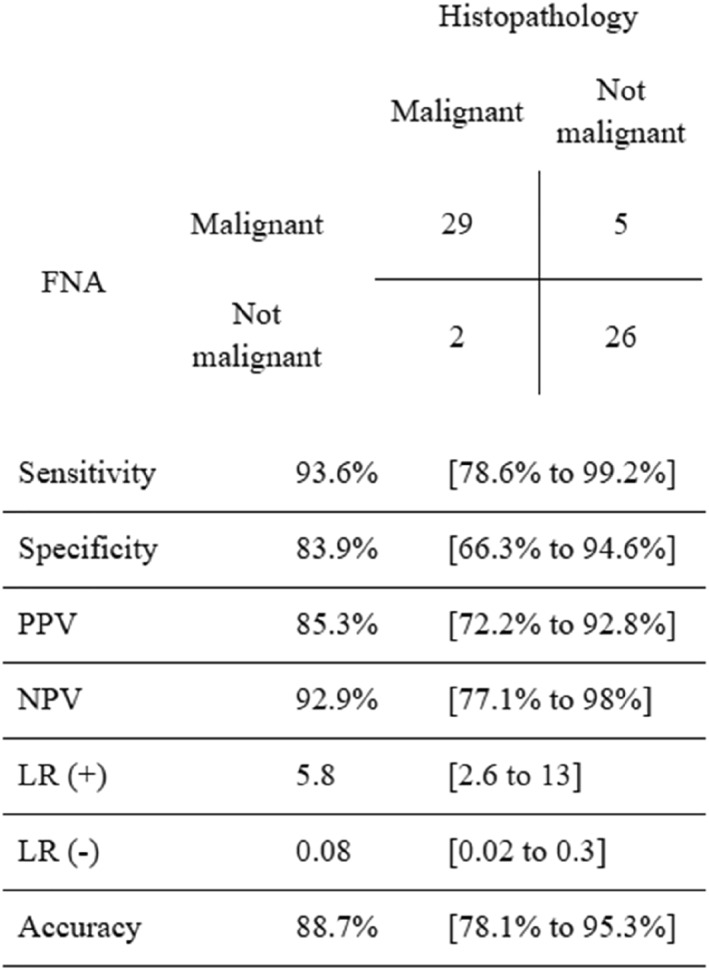
Predictive values of fine‐needle aspiration for diagnosing soft tissue and bone malignancies in salivary glands (*n* = 62).

The MSRSGC achieved a sensitivity of 93.6%, specificity of 83.9%, positive predictive value of 85.3%, negative predictive value of 92.9%, and diagnostic accuracy of 88.7% in predicting soft tissue and bone malignancies of the salivary glands (Figure 1).

**FIGURE 2:**
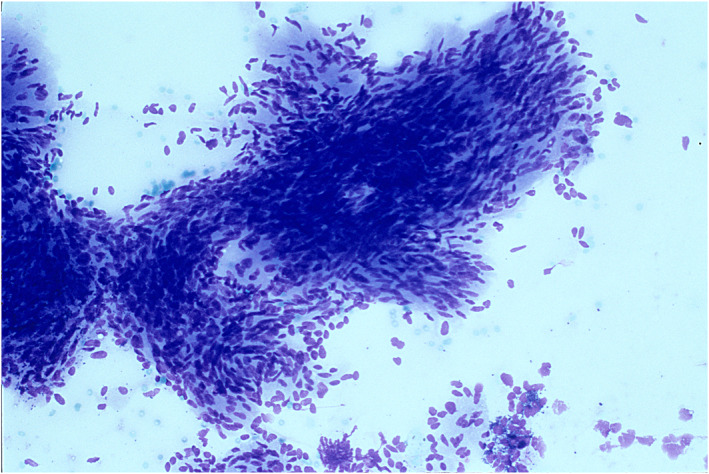
Schwannoma. Spindle cells without cyto‐nuclear atypia and scant fibrillary background.

**FIGURE 3:**
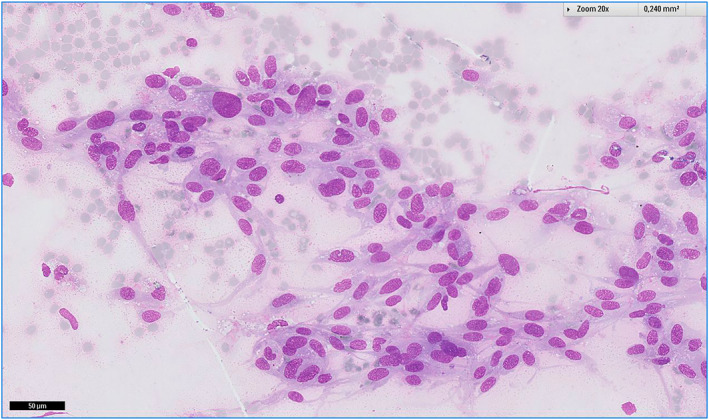
Polymorphous sarcoma composed of spindle malignant cells.

**FIGURE 4:**
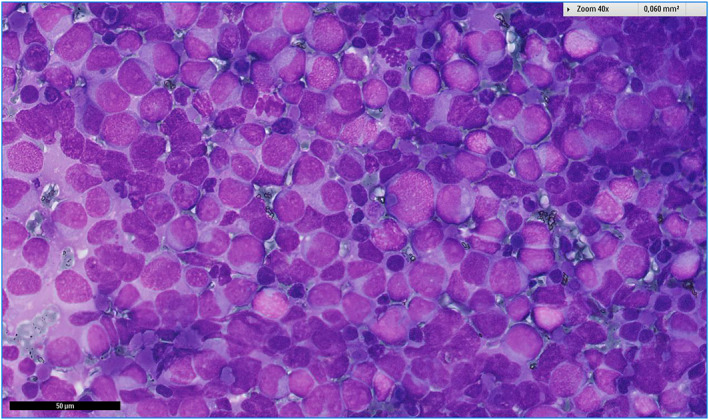
Metastatic rhabdomyosarcoma composed of roundish and epithelioid cells.

**FIGURE 5:**
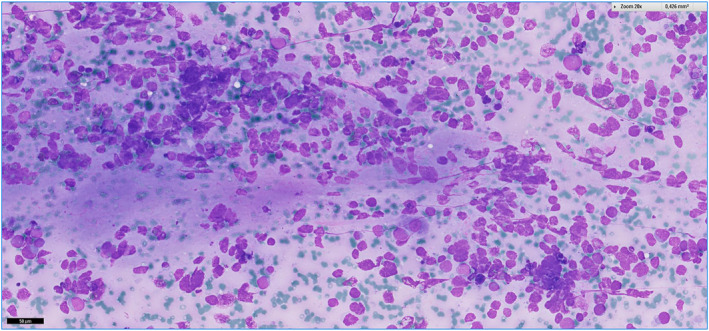
Metastatic neuroblastoma. Atypical neuroblasts and delicate background neuropil.

Examples of cases are illustrated in Figures 2, 3, 4, 5. FNA correctly identified the nonmalignant nature of the tumors in 36 of 42 cases (85.7%). In 21 of 42 cases (50%), the initial cytologic diagnosis exactly matched the final diagnosis (Table 7). The benign tumors with the highest detection rates were lipoma (81.3%), hemangioma (66.7%), paraganglioma, fibroma, granular rhabdomyoma, and neurofibroma (all cases). Conversely, schwannoma (zero of four), myopericytoma (zero of two), nodular fibrosis (zero of two), and neuroma (zero of two) presented significant diagnostic challenges because none were diagnosed with full accuracy by cytology. In 21 cases (50%), we observed discrepancy between the cytologic and final diagnosis. Of these, six were considered nonneoplastic, including five cases showing only normal cells (subsequently diagnosed as two lipomas, one schwannoma, one fibromatosis, and one neuroma) and one case with inflammation, later confirmed as a lipoma. Nine cases were misclassified: three myoepitheliomas were diagnosed as pleomorphic adenomas, two schwannomas as pleomorphic adenomas, one fibromatosis as a conjunctival tumor, one hemangioma as fusiform cells (suggestive of a benign neoplasm), one myopericytoma as a fibroma, and one vascular hamartoma as a hemangioma. Six cases were upscored. Four were initially classified as SM: one myoepithelioma was diagnosed as adenoid cystic carcinoma, one schwannoma as a sample suspicious for malignancy, one fibromatosis as sarcoma, and one nodular fasciitis as an atypical conjunctival tumor. Two cases were initially classified as malignant: one myopericytoma was diagnosed as fusiform tumor cells and one nodular fasciitis as a spindle cell tumor.

**TABLE 6:** Comparison of ROM between the classic European system, the Milan System, and the IAC–IARC–WHO System categories.

Classic system	ROM (%)	Milan System	ROM (%)	IAC–IARC–WHO System	ROM (%)
Not significant	—	Nondiagnostic	—	Insufficient/inadequate/nondiagnostic	—
Benign	7.1	Nonneoplastic	25	Benign	7.1
		Benign	0		
Suspicious/inconclusive	16.7	AUS	—	Atypical	—
		SUMP	0	STNUMP	0
		SM	20	SM	20
Malignant	96.6	Malignant	96.6	Malignant	96.6

Abbreviations: AUS, atypia of undetermined significance; IAC–IARC–WHO, International Academy of Cytology‐International Agency for Research on Cancer‐World Health Organization; ROM, risk of malignancy; SUMP, salivary gland neoplasm of uncertain malignant potential; STNUMP, soft tissue neoplasm of uncertain malignant potential; SM, suspicious for malignancy.

**TABLE 7:** Nonmalignant soft tissue and bone tumors of the salivary glands.

Final diagnosis^a^	No. (%)	Histological follow‐up (%)	No. of cases	Cytological diagnosis	Milan score	Diagnosed by FNA (%)	Lack of malignancy detected by FNA (%)
Lipoma	16 (38.1)	9 (56.25)	13	Lipoma	IVa	13/16 (81.25)	16/16 (100)
			2	Normal cells	II		
			1	Inflammation	II		
Myoepithelioma	5 (11.9)	4 (80)	3	Pleomorphic adenoma	IVa	1/5 (20)	4/5 (80)
			1	Myoepithelioma	IVa		
			1	Adenoid cystic carcinoma	V		
Schwannoma	4 (9.5)	4 (100)	2	Pleomorphic adenoma	IVa	0/4 (0)	3/4 (75)
			1	Suspicion of malignancy	V		
			1	Normal cells	II		
Fibromatosis	3 (7.1)	3 (100)	1	Normal cells	II	0/3 (0)	2/3 (66.7)
			1	Conjunctival tumor	IVa		
			1	Sarcoma, NOS	V		
Hemangioma	3 (7.1)	2 (66.7)	2	Hemangioma	IVa	2/3 (66.7)	3/3 (100)
			1	Fusiform cells	IVa		
Myopericytoma	2 (4.8)	2 (100)	1	Fibroma	IVa	0/2 (0)	1/2 (50)
			1	Fusiform tumor cells	VI		
Nodular fasciitis	2 (4.8)	2 (100)	1	Myxoid spindle cell tumor	VI	0/2 (0)	0/2 (0)
			1	Atypical conjunctival tumor	V		
Paraganglioma	2 (4.8)	1 (50)	2	Paraganglioma	IVa	2/2 (100)	2/2 (50)
Fibroma	1 (2.4)	1 (100)	1	Fibroma	IVa	1/1 (100)	1 (100)
Granular rhabdomyoma	1 (2.4)	1 (100)	1	Granular rhabdomyoma	IVa	1/1 (100)	1 (100)
Neurofibroma	1 (2.4)	0 (0)	1	Neurofibroma	IVa	1/1 (100)	1 (100)
Neuroma	1 (2.4)	1 (100)	1	Normal cells	II	0/1 (0)	1 (100)
Vascular hamartoma	1 (2.4)	1 (100)	1	Hemangioma	IVa	0/1 (100)	1 (100)

Abbreviations: FNA, fine‐needle aspiration; NOS, not otherwise specified.

^a^ In selected cases, final diagnoses were supported by immunohistochemistry and/or molecular studies, in addition to histopathological evaluation.

The accuracy of cytological diagnosis was higher for malignant tumors than for benign ones, with cytological diagnoses exactly matching the final diagnoses in 30 of 44 cases (68.2%). Notably, FNA accurately diagnosed malignant soft tissue and bone salivary gland tumors in 42 of 44 cases (95.5%), with exceptions being one case each of metastatic leiomyosarcoma and metastatic osteosarcoma (Table 8). FNA analysis precisely identified nine of 13 metastatic rhabdomyosarcomas (69.2%), two of four primary rhabdomyosarcomas (50%), four of five sarcomas (80%), four of four metastatic neuroblastomas (100%), and three of four chordomas (75%). No cases of malignant peripheral nerve sheath tumor, metastatic chondrosarcoma, or metastatic sarcoma were identified by FNA (one case each). Of the 14 cases in which the FNA diagnosis did not exactly match the final diagnosis, nine were initially categorized as malignant tumors of other histology, two were unspecified neoplastic cells, one was suspicious for malignancy, one was clinically suspicious for malignancy but showed cytological features characteristic of nonneoplastic cells, and one was considered nonneoplastic.

**TABLE 8:** Malignant soft tissue and bone tumors involving salivary glands.

Final diagnosis^a^	No. (%)	Histological follow‐up (%)	No. of cases	Cytological diagnosis (*n*)	Milan score	Diagnosis by FNA (%)	Malignancy detected by FNA (%)
Metastatic rhabdomyosarcoma	13 (29.5)	8 (61.5)	6	Metastatic rhabdomyosarcoma	VI	9/13 (69.2%)	13 (100)
			3	Rhabdomyosarcoma	VI		
			3	Sarcoma, NOS	VI		
			1	Neoplastic cells	VI		
Sarcoma, NOS	5 (11.4)	4 (80)	3	Sarcoma, NOS	VI	4/5 (80%)	5 (100)
			1	Metastatic sarcoma, NOS	VI		
			1	Malignant hemangioma	VI		
Metastatic chordoma	4 (9.1)	3 (75)	3	Chordoma	VI	3/4 (75)	4 (100)
			1	Neoplastic cells	VI		
Metastatic neuroblastoma	4 (9.1)	0 (0)	4	Neuroblastoma	VI	4/4 (100)	4 (100)
Primary rhabdomyosarcoma	4 (9.1)	4 (100)	2	Rhabdomyosarcoma	VI	2/4 (100)	4 (100)
			1	Conjunctival tumor	VI		
			1	Sarcoma, NOS	VI		
Adamantinoma	3 (6.8)	2 (66.7)	2	Adamantinoma	VI	2/3 (66.7)	3/3 (100)
			1	Suspicion of malignancy	V		
Metastatic leiomyosarcoma	3 (6.8)	2 (66.7)	2	Leiomyosarcoma	VI	2/3 (66.7)	2/3 (66.7)
			1	Suspicion of malignancy	II		
Metastatic osteosarcoma	2 (4.5)	2 (100)	1	Osteosarcoma	VI	1/2 (50)	1/2 (50)
			1	Normal cells	II		
Angiosarcoma	1 (2.3)	1 (100)	1	Angiosarcoma	VI	1/1 (100)	1 (100)
Dermatofibrosarcoma protuberans	1 (2.3)	1 (100)	1	Dermatofibrosarcoma protuberans	VI	1/1 (100)	1 (100)
Fibrosarcoma	1 (2.3)	1 (100)	1	Fibrosarcoma	VI	1/1 (100)	1 (100)
Malignant Peripheral Nerve Sheath Tumor	1 (2.3)	1 (100)	1	Polymorphic carcinoma	VI	0/1 (0)	1 (100)
Metastatic chondrosarcoma	1 (2.3)	1 (100)	1	Sarcoma, NOS	VI	0/1 (0)	1 (100)
Metastatic sarcoma, NOS	1 (2.3)	1 (100)	1	Sarcoma, NOS	VI	0/1 (0)	1 (100)

Abbreviations: FNA, fine‐needle aspiration; NOS, not otherwise specified.

^a^ In selected cases, final diagnoses were supported by immunohistochemistry and/or molecular studies, in addition to histopathological evaluation.

## DISCUSSION

Soft tissue and bone tumors of the salivary glands constitute a rare and diverse group characterized by complex clinical course and diagnostic challenges. Although the MSRSGC was originally not intended for evaluating soft tissue and bone tumors, it accurately identified 95.5% (42 of 44) of malignant cases. Overall, it achieved high accuracy (88.3%), sensitivity (93.6%), and specificity (82.7%) in detecting malignancies, underscoring its clinical utility in guiding decisions regarding surgical intervention. Notably, malignant tumors were more prevalent among younger patients compared to benign cases (median age, 21 vs. 42 years), emphasizing the importance of auxiliary studies in this demographic subset. It should also be noted that the Milan System includes nonsalivary neoplasms, such as melanoma and lymphoma, when they present as salivary gland tumors.^12^

The ROM analysis showed similar results between the 4‐tier European System and the IAC–IARC–WHO System, which can be attributed to the lack of inconclusive results in our cohort (Tables 3 and 5). On the other hand, ROM calculated for the Milan System categories was slightly higher for the ND category and lower for the BN categories, which was caused by the subdivision of categories containing a small number of cases and the exclusion of the NN cases (Table 6). For this reason the ROM may have been inflated and not accurately reflect the real‐world prevalence of soft tissue and bone tumor of the salivary glands. Hence, the classic system and the IAC–IARC–WHO System may be more suitable for estimating ROM in small cohorts.

False‐positives were more frequent than false‐negatives and often involved peripheral nerve sheath tumors and tumors of uncertain differentiation. On the other hand, both false‐negatives—metastatic osteosarcoma and metastatic leiomyosarcoma—are typically not diagnosed by routine cytology and necessitate additional diagnostic methods.

Lipoma was the most common tumor in our cohort, comprising 38.1% (16 of 86) of the cases and representing the only adipocytic tumor. FNA was sufficient to diagnose lipoma in 13 of 16 cases (81.3%), with no initial misclassification as malignant (Table 7). Of the three misdiagnosed samples, two showed only normal salivary gland cells and one exhibited inflammatory changes. In adipocytic lesions such as lipomas, cytologic findings may overlap with normal salivary gland fat or fatty degeneration and often require histopathologic confirmation for definitive diagnosis. The prevalence of lipoma in other studies ranges from 5.3% (one of 19) to 56% (38 of 68) of all mesenchymal tumors of the salivary glands, and it typically does not pose significant diagnostic challenges.^14^, ^15^, ^16^ It is also worth noting that, in cases of lipoma, the presence of normal cells on FNA may be compatible with a benign adipocytic lesion, but correlation with clinical and radiologic findings remains essential for accurate diagnosis. Although 66.7% (two of three) of hemangiomas in our study were correctly diagnosed by cytology, this tumor is frequently overlooked. For instance, the only hemangioma identified by Isgor et al.^17^ was deemed nondiagnostic, and both nondiagnostic results in the Bhrarti et al.^18^ study were later confirmed as hemangiomas.

Other benign mesenchymal tumors were more challenging to diagnose. No schwannoma, the most common benign neural tumor of the salivary glands, was correctly diagnosed by cytology. However, three of four cases were initially interpreted as benign neoplasms, (two pleomorphic adenomas and one normal salivary gland sample), whereas one was classified as suspicious for malignancy (Table 7). These findings are consistent with the known diagnostic difficulties in differentiating schwannomas from other salivary gland tumors.^3^, ^19^ Schwannomas can be confused with pleomorphic adenomas or myoepitheliomas, with some pleomorphic adenomas being particularly difficult to differentiate. Furthermore, schwannomas with nuclear atypia can be mistaken for sarcomas.^2^

Myoepitheliomas often present cytologic features that overlap with malignant tumors, typically leading to classification as SUMP on FNA.^2^, ^20^ Other classifications are not uncommon; for example, in the study by Jha et al.,^21^ only one myoepithelioma was nondiagnostic, whereas Maleki et al.^3^ accurately diagnosed it as a benign neoplasm. Nodular fasciitis is rarely identified by cytology and is often considered in the differential diagnosis of pleomorphic adenomas, which can also resemble schwannomas or hemangiomas due to their spindled or palisading morphology with bland nuclear features.^1^, ^2^

Fibromatosis is a locally aggressive neoplasm that does not metastasize. Although FNA is commonly used to diagnose it, fibromatosis may resemble low‐grade spindle cell sarcomas on cytology and requires histological confirmation. The clinical behavior of fibromatosis is usually reflected in its cellularity, with aspirates from aggressive fibromatosis being more cellular, though mitoses are rare to absent.^22^, ^23^

Malignant soft tissue and bone tumors of the salivary glands were easier to diagnose by FNA than benign tumors, with nearly all cases accurately assessed as malignant (42 of 44 cases). The high proportion of metastatic tumors (28 of 44 malignant cases, 63.6%) reflects the referral profile of our institution, where FNAs frequently target parotid‐region lymph nodes or adjacent soft tissue masses in patients with known or suspected primary malignancies. In line with real‐world clinical practice, cytologic evaluation incorporated available clinical information, including a history of prior malignancy, which may have contributed to diagnostic accuracy.

Rhabdomyosarcoma, including four primary and 13 secondary cases, was the most common malignancy in our cohort, representing 2.1% of all salivary gland malignancies (17 of 823). It was also a common finding in the study by Maleki et al.,^3^ accounting for 13.9% of all malignancies (11 of 79). Notably, the malignant nature of all 17 cases diagnosed at our institution was accurately identified during cytological assessment, suggesting that finding primitive, round, or spindle cells showing skeletal muscle differentiation strongly indicate rhabdomyosarcoma.^24^ However, identifying specific subtypes often requires histopathological examination.

Neuroblastoma was the only malignancy consistently recognized by FNA.^3^ In our study, all four cases were diagnosed solely by FNA without the need for additional testing. The only malignancies misclassified as benign by FNA were metastatic leiomyosarcoma and metastatic osteosarcoma. Differentiating between leiomyoma and leiomyosarcoma is complex and relies on the mitotic count, necessitating histopathological examination.^22^ Aspirates usually have low cellularity, with necrosis suggesting underlying malignancy.^25^ In our study, the false‐negative leiomyosarcoma case showed no cytological features of malignancy, earning an MSRSGC category of NN, but was suspicious for malignancy based on clinical examination, prompting histopathological follow‐up. The same approach was taken for the missed osteosarcoma.

In conclusion, despite their rarity, soft tissue and bone tumors of the salivary glands can be effectively diagnosed by FNA, with exceptionally high accuracy for malignant cases. FNA demonstrated high accuracy in the BN category but moderate reliability in distinguishing NN cases, where a 25% malignancy rate was observed. Caution is warranted with fibroblastic, myofibroblastic, and peripheral nerve sheath tumors because their spindle‐shaped cells present diagnostic challenges in cytology. The clinical utility of the MSRSGC categories AUS and SUMP in diagnosing soft tissue and bone tumors of the salivary glands requires further validation. Comparative analysis demonstrated that all three systems—the classic European 4‐tier system, the MSRSGC, and the IAC–IARC–WHO System—showed similar diagnostic performance in malignant cases. Minor differences were observed in benign and indeterminate categories, highlighting the need for tailored application depending on cohort size and tumor type. Continued refinement of diagnostic criteria and a multidisciplinary approach that integrates clinical, imaging, and cytological data are essential for optimal patient management.

## AUTHOR CONTRIBUTIONS

**Adam Kowalewski**: Conceptualization; investigation; methodology; validation; and writing—original draft. **Jędrzej Borowczak**: Conceptualization and writing—original draft. **Hervé J. Brisse**: Conceptualization; investigation; methodology; validation. **Olivier Choussy**: Conceptualization and writing— original draft. **Jerzy Klijanienko**: Conceptualization, investigation; methodology; validation; writing— original draft; writing—review and editing; project administration; supervision; visualization.

## CONFLICT OF INTEREST STATEMENT

Jerzy Klijanienko reports consulting fees from Université de Recherche Paris Sciences et Lettres. The other authors declare no conflicts of interest.
